# Genome-wide impacts of alien chromatin introgression on wheat gene transcriptions

**DOI:** 10.1038/s41598-020-61888-1

**Published:** 2020-03-16

**Authors:** Zhenjie Dong, Chao Ma, Xiubin Tian, Changtao Zhu, Gang Wang, Yuanfeng Lv, Bernd Friebe, Huanhuan Li, Wenxuan Liu

**Affiliations:** 1 National Key Laboratory of Wheat and Maize Crop Science, College of Life Sciences, Henan Agricultural University, Zhengzhou, 450002 China; 2 Yu’An Institute of Wheat, Wen County, Jiaozuo, 454850 China; 3 Wheat Genetic Resources Center, Department of Plant Pathology, Throckmorton Plant Sciences Center, Kansas State University, Manhattan, KS 66506 USA

**Keywords:** Plant molecular biology, Plant genetics

## Abstract

Agronomic characteristics and tolerance to biotic and abiotic stresses in hexaploid wheat can be drastically improved through wheat-alien introgression. However, the transcriptional level interactions of introduced alien genes in the wheat genetic background is rarely investigated. In this study, we report the genome-wide impacts of introgressed chromosomes derived from *Ae. longissima* on gene transcriptions of the wheat landrace Chinese Spring. RNA-seq analyses demonstrated 5.37% and 4.30% of the genes were significantly differentially expressed (DEGs) in CS-*Ae*. *longissima* disomic 3S^l^#2(3B) substitution line TA3575 and disomic 6S^l^#3 addition line TA7548, respectively when compared to CS. In addition, 561 DEGs, including 413 up-regulated and 148 down-regulated or not transcribed genes, were simultaneously impacted by introgressed chromosomes 3S^l^#2 and 6S^l^#3, which accounts for 41.25% of the DEGs in TA3575 and 38.79% in TA7548. Seventeen DEGs, annotated as R genes, were shared by both introgression lines carrying chromosomes 3S^l^#2 and 6S^l^#3, which confer resistance to powdery mildew. This study will benefit the understanding of the wheat gene responses as result of alien gene(s) or chromosome intogression and the plant defense response initiated by powdery mildew resistance genes in chromosomes 3S^l^#2 and 6S^l^#3.

## Introduction

Allohexaploid wheat (*Triticum aestivum* L., 2*n*** =** 6*x*** =** 42, AABBDD) is a species of the genus *Triticum* in the grass family *Poaceae* (or *Gramineae*). Wheat evolved from two independent natural hybridization events followed by chromosome doubling in the new hybrids. The first hybridization occurred between *Triticum urartu* Tumanian ex Gandilyan (2*n*** =** 2*x*** =** 14, AA) and *Aegilops speltoides* Tausch (2*n*** =** 2*x*** =** 14, SS) about 0.5 million years ago, forming a new tetraploid species *T. turgidum* L. (2*n*** =** 4*x*** =** 28, AABB)^1,2^. The domesticated *T. turgidum* subsp. *dicoccum* then hybridized with another diploid species, *Ae*. *tauschii* Coss. (2*n*** =** 2*x*** =** 14, DD) about 7,000 to 12,000 years ago, producing a fertile hexaploid bread wheat (2*n*** =** 6*x*** =** 42, AABBDD)^3–7^. Although of relatively recent origin, hexaploid bread wheat is currently the most favored staple food for human beings planted on the most acres and most traded food crop in the world.

Although common wheat is one of the earliest domesticated crop plants, it is still a young species compared with its ancestors or other wild relatives. Gene pools derived from wild relatives, which have a much longer evolutionary history, play an important role in modern wheat improvement. For example, stem rust, caused by *Puccinia graminis* f. sp. *tritici* (*Pgt*), was a devastating disease during the 20^th^ century. However, epidemics of stem rust were well under control in the past several decades largely due to the deployment of highly effective resistance genes, such as *Sr31* from *Secale cereale* L., *Sr24* from *Thinopyrum elongatum* (Host) D. R. Dewey, and *Sr36* from *T. timopheevii* (Zhuk.) Zhuk. in wheat varieties worldwide. However a novel race named Ug99 (TTKSK) emerged in Uganda (1999) having virulence for *Sr31* and many other stem rust resistance genes thus Ug99 has become a major threat to wheat production^8–13^. Continuous efforts are needed to identify and deploy new, effective and durable resistance genes to maintain world food security. Of the currently catalogued genes against the major diseases in wheat like leaf rust, stripe rust, and powdery mildew, at least 50% of those genes have been derived from wheat ancestors or other wild relatives including *Ae. tauschii*, *Ae*. *speltoides*, *Ae*. *geniculata* Roth, *Ae*. *longissima* Schweinf. & Muschl., *S. cereale*, *Dasypyrum villosum*, *Th. intermedium* (Host) Barkworth & D. R. Dewey, *Th. elongatum*, and *Agropyron cristatum* (L.) Gaertn^14,15^.

Despite the importance of the improvement of genetic diversity in common wheat, most alien genes are currently under-utilized due to the limitation of deleterious linkage drag introduced with alien chromosome segments. Linkage drag could be decreased by either shortening the targeted gene segments or possibly deactivating the undesired deleterious genes. For example, the wheat-rye T1BL·1RS translocation has been widely used in wheat cultivars or advanced breeding lines from Europe, China, India, the USA, and other developing countries since the mid-1980s because of remarkable agronomic and disease-resistant characteristics. The short arm of chromosome 1 R (1RS) from *S. cereale* not only carries resistance genes against fungal pathogens, such as stripe rust resistance gene *Yr*9, leaf rust resistance gene *Lr*26, stem rust resistance gene *Sr*31 and *Pm*8 against wheat powdery mildew^16–19^, but also enhances root biomass, grain yield, protein content^20–23^ and tolerance to drought^24–26^. However, chromosome arm 1RS also carries *Sec*-1 loci encoding γ-secalin and ω-secalin high molecular weight proteins, which decreases the processing and baking quality of wheat containing the T1BL·1RS translocation^27–30^.

In addition, linkage drag may also be caused by the inability of introduced alien chromosome segments to adequately compensate for the loss of their homoeologous counterparts or incompatible gene interactions between the alien donor and the recipient wheat line. Parallel with the recent rapid progress of genome sequencing techniques and ever increasing evidence from genome sequence analyses has shown that linkage drag maybe a result of gene expression/suppression caused by gene interactions between the donor and the recipient, post-translational modifications of gene products, and heteromeric complexes formed by the donor and the recipient gene products^31–35^. However, only a few investigations of chromosome-wide gene expression in alien chromosome addition lines have been reported^36–38^. Rey *et al*. (2018) reported that the ditelosomic addition of 7HL from *Hordeum vulgare* L. into the genetic background of CS could lead to 3% differentially expressed genes (DEGs) of wheat and 42% of the 7HL-derived genes in the wheat-barley 7HL addition line^38^. Nevertheless, the regulatory mechanism underlying the widespread gene transcription of the alien chromosome(s) is still unclear.

*Aegilops longissima* (2*n* = 2*x* = 14, S^l^S^l^) is an S-genome diploid species belonging to the section *Sitopsis*^3^. This species contains many traits desirable for wheat improvement, such as the powdery mildew resistance gene *Pm13* located on chromosome 3S^l^, which has been widely used in wheat breeding programs^39,40^. In addition, we have previously reported that chromosome 6S^l^#3 (TA7548) also confers resistance to wheat powdery mildew^41^. Several QTLs conferring resistance to wheat eyespot are located on chromosomes 1S^l^, 3S^l^, 5S^l^ and 7S^l^^42,43^. Additionally, chromosome 1S^l^ has been reported to carry genes enhancing bread-making quality^44,45^. A few wheat-*Ae*. *longissima* amphiploids and sets of addition and substitution lines have been developed and several translocation lines, such as T3BL·3BS-3S^l^ and T3DL·3DS-3S^l^ translocations carrying *Pm13*, have been deployed in wheat breeding programs worldwide^39,40,44,46–49^.

In this study, we report the genome-wide impact on gene transcription in a recipient wheat landrace CS caused by the introgression of pairs of the alien chromosomes 3S^l^#2 and 6S^l^#3 (*Ae*. *longissima*), each of which harbors resistance genes to powdery mildew^41^, based on comparative transcriptome analyses of CS with a CS-*Ae*. *longissima* disomic 3S^l^#2(3B) substitution line (TA3575) and a disomic 6S^l^#3 addition line (TA7548).

## Results

### Karyotype analysis of TA3575

**Figure 1:**
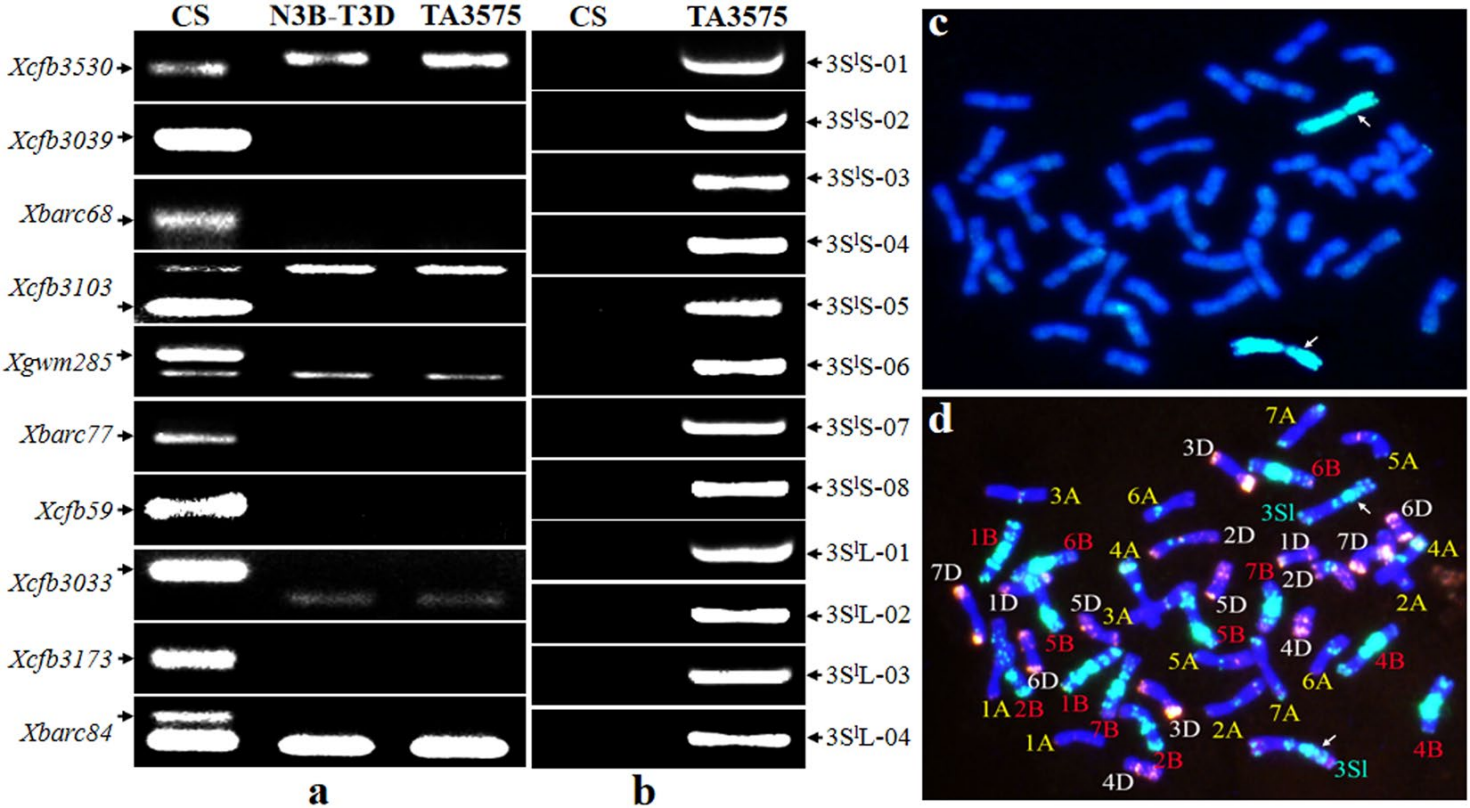
Karyotype analysis of TA3575 using molecular markers and *in situ* hybridization. (**a**) Electrophoresis patterns of molecular markers of wheat chromosome 3B-specific. N3B-T3D represents Nullisomic 3B-Tetrasomic 3D. **(b)**. Electrophoresis patterns of molecular markers of 3S^l^#2 from *Ae*. *longissima*. Arrows show the specific amplification bands of chromosome 3B (**a**) and 3S^l^#2 (**b**). (**c**) GISH patterns of TA3575, chromosomes 3S^l^#2 were painted in green and wheat chromosomes in blue. (**d)** FISH patterns of TA3575. Signals from oligos pAs1-3, pAs1-4, pAs1-6, AFA-3, AFA-4 and (AAC)_10_ displayed in red, those from oligos pSc119.2-1 and (GAA)_10_ in green, and chromosomes of wheat were stained with DAPI displaying in blue. Arrows indicated chromosome 3S^l^#2.

Karyotype analysis of TA3575 was identified by integration analyses of PCR amplification using ten 3B-specific and 12 3S^l^-specific markers (Supplementary Table S1) and *in situ* hybridization of root-tip cell mitotic metaphase chromosomes. The PCR results showed that no 3B-specific bands were amplified using 3B-specific primer pairs in both TA3575 and the N3B-T3D control, where a pair of chromosome 3B was replaced by two pairs of 3D. Conversely the 3S^l^#2-specific amplifications resulted only from TA3575, indicating the absence of 3B chromosomes and the presence of alien chromosome 3S^l^#2 in TA3575. The *in situ* hybridization results confirmed that in TA3575, 20 pairs of wheat chromosomes plus a pair of 3S^l^#2 chromosomes from *Ae. longissima* replaced the pair of 3B chromosomes (Fig. 1, Supplementary Fig. S1). Thus, TA3575 was redesignated as a CS-*Ae. longissima* disomic substitution (DS) 3S^l^#2(3B) line, rather than the disomic addition (DA) 3S^l^#2 line previously identified by the Wheat Genetic Resource Center (WGRC), Kansas State University, Manhattan, KS.

### RNA-seq quantity analysis, sequence assemble

A total of 77,962,967, 77,492,142 and 76,266,826 clean reads (>100 bp) were obtained from RNA sequencing for CS, TA3575 and TA7548, respectively. Sequencing quality scores Q30, which inferred a base call accuracy of 99.9%, were greater than 93.06% for all samples, which signifies that the RNA-seq quality was appropriate for subsequent sequence assembly and analysis.

Clean reads of all the samples were further assembled into a total of 158,953 unigenes with a median length of 1,247.95 bp using the short-read assembly software, Trinity. The contig N50 value was 1,725 bp in length. We mapped 91.29% clean reads back to the reference assembly.

### Gene expression analysis

Expression levels of the 158,953 genes were normalized based on fragments per kilobase per million mapped reads (FPKM) values of the sequences for further analysis of differential genes. A total of 69,808 genes were transcribed by CS at an FPKM ≥ 0.5 in two independent biological replicates, of which 37,058 (53.10%) were assigned to the wheat genome (expect < =1e-10&qcov > =75%) (Supplementary Tables S2, S3). Pairwise comparison of expression of 37,058 mapped-genes in CS with the CS-*Ae. longissima* DS 3S^l^#2(3B) line TA3575 and CS-*Ae*. *longissima* DA 6S^l^#3 line TA7548 yielded 1,991 (5.37%) and 1,592 (4.30%) genes, respectively, which were significantly differentially transcribed with a cut-off of |log_2_(fold change)| ≥ 1 and P < 0.05. These differential genes are described as differentially expressed genes (DEGs) for convenience. Expression pattern analyses revealed that the majority of the DEGs were non-expressed genes (NEGs) or down-regulated expressed genes (DRGs) in both TA3575 (1,253/1,991, 62.93%) and TA7548 (922/1,592, 57.91%). Up-regulated expressed genes (URGs) accounted for 37.07% in TA3575 and 42.09% in TA7548 (Table 1, Supplementary Tables S2–S4, Supplementary Fig. S2).

**Table 1:** Comparison of transcription changed genes in TA3575 and TA7548.

Lines	Group No.	NEGs	DRGs	URGs	DEGs	(NEGs + DRGs) %	URGs %	(DEGs/CS) %	Note
**TA3575**	G1	59	19	97	175	44.57	55.43	3.71	
	G2	91	39	116	246	52.85	47.15	4.26	
	G3	511	129	141	781	81.95	18.05	14.50	
	G4	58	19	97	174	44.25	55.75	3.63	
	G5	62	17	103	182	43.41	56.59	3.28	
	G6	65	26	101	192	47.40	52.60	4.01	
	G7	69	23	75	167	55.09	44.91	3.11	
	unChr*	42	24	8	74	89.19	10.81	10.83	
	Subtotal1	957	296	738	1991	62.93	37.07	5.37	3B included
	Subtotal2	496	196	722	1414	48.94	51.06	4.01	3B excluded
**TA7548**	G1	41	61	89	191	53.40	46.60	4.05	
	G2	60	60	110	230	52.17	47.83	3.98	
	G3	46	72	96	214	55.14	44.86	3.97	
	G4	42	39	79	160	50.63	49.38	3.34	
	G5	48	49	107	204	47.55	52.45	3.68	
	G6	93	166	100	359	72.14	27.86	7.50	
	G7	62	48	82	192	57.29	42.71	3.58	
	unChr*	4	31	7	42	83.33	16.67	6.15	
	Subtotal1	396	526	670	1592	57.91	42.09	4.30	6A included
	Subtotal2	368	466	665	1499	55.64	44.36	4.20	6A included

*unChr: genetic group was not assigned.

### Distribution of DEGs in TA3575

Analyzing the chromosome distribution of 37,058 mapped-genes in CS demonstrated that these genes were unevenly located across all 21 wheat chromosomes (CS RefSeq v1.0, IWGSC 2018). Of the 21 chromosome pairs, chromosome 2B possessed a maximum number of transcribed genes (2,066/37,058, 5.58%), followed by 5B (2,035, 5.49%), 2D (1,937, 5.23%) and 6B (1,931, 5.21%), whereas the minimum number of genes were mapped on chromosome 6A (138, 3.72%) (Supplementary Table S2).

Similarly, the 1,991 DEGs in TA3575 also covered all seven wheat homoeologous groups and but were unevenly distributed (Table 1) like in CS. The most DEGs were located on group 3 of CS, where the transcription of 781 (14.50%) out of 5,387 genes were significantly affected by the introgression of 3S^l^#2 replacing chromosome 3B. Of those 781 DEGs, 511 (65.43%) and 129 (16.52%) genes were non-transcribed and down-regulated, respectively, whereas the remaining 141 genes (18.05%) were up-regulated. In contrast, the transcription of genes in group 7 was least affected by introgression of 3S^l^#2, and only 1.83% (98/5,369) of the genes were differentially transcribed (Table 1).

**Figure 2:**
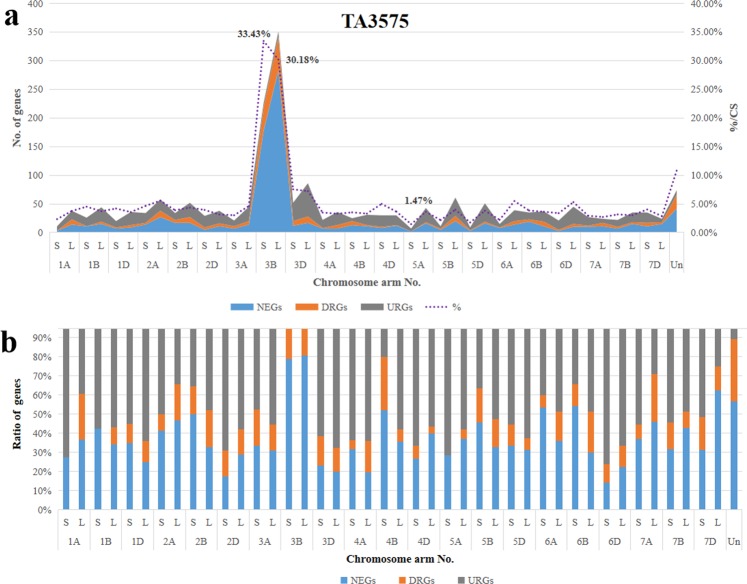
Chromosome distribution of DEGs in TA3575. (**a)** Distribution of DEGs on different chromosome arms. (**b**) Ratios of different DEGs in different chromosomes.

Further analysis of DEG distribution on wheat chromosomes showed that most DEGs mapped to wheat chromosome 3B; 577 (31.43%) out of the 1,839 genes mapped on chromosome 3B in CS were differentially transcribed in TA3575, with 33.43% on the short and 30.18% on the long arm of chromosome 3B (Fig. 2). Analysis of the expression pattern of these 577 DEGs showed that the majority (461, 79.90%) were non-transcribed and 100 genes (17.33%) were down-regulated. Only 16 (2.77%) DEGs were up-regulated, indicating that at least 34.57% (461/1,839) of genes on the missing chromosome 3B were not genetically compensated by the introgressed 3S^l^#2 chromosome from *Ae*. *longissima*, regardless of gene expression level differences. The DEGs from others chromosome-arms averaged 4.01% (1,413/35,219), ranging from 1.47% (7/475) for the short arm of 5 A (5AS) to 7.23% (86/1,190) for the long arm (5AL) and 7.55% (52/689) for the short arm of chromosome 3D (Fig. 2). Major transcription pattern changes were different for different wheat chromosomes. Besides chromosome 3B, 80% of the DEGs for chromosome 4BS were non-transcribed or down-regulated genes (Fig. 2), whereas, for chromosome 6DS, at least 75% of the DEGs were up-regulated in the presence of chromosome 3S^l^#2 in TA3575 (Fig. 2).

### Validation of differentially transcribed genes in TA3575

**Figure 3:**
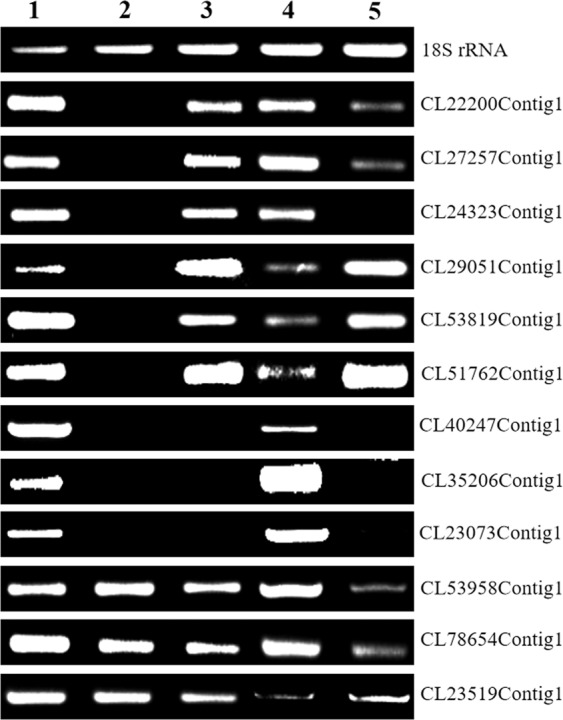
Validation of differentially transcribed genes in TA3575 by PCR amplification of gDNA and semi-quantitative RT-PCR. 1. gDNA of CS; 2. gDNA of N3B-T3D; 3. gDNA of TA3575; 4. cDNA of CS; 5. cDNA of TA3575.

We selected nine genes mapped to chromosome 3B and three genes from other chromosomes to validate gene transcription changes in TA3575 using 18S rRNA as a control (Supplementary Table S1). PCR amplification using genomic DNA (gDNA) from control CS, N3B-T3D, and TA3575 as templates showed that all nine 3B genes were present in CS and absent in N3B-T3D, as expected. However, in TA3575, six of the nine 3B-specific genes, including CL22200Contig1, CL27257Contig1, CL24323Contig1, CL29051Contig1, CL53819Contig1 and CL51762Contig1, were amplified and the remaining three (CL40247Contig1, CL35206Contig1 and CL23073Contig1) were absent (Fig. 3, Supplementary Fig. S3). These results indicate that the six 3B-specific sequences were also present on introgressed *Ae*. *longissima* chromosome 3S^l^#2, whereas the other three were absent on chromosome 3S^l^#2. Using the non-3B-specific primer pairs (CL53958Contig1, CL78654Contig1, and CL23519Contig1), gDNA of CS, N3B-T3D, and TA3575 produced the expected PCR amplification (Fig. 3, Supplementary Fig. S3).

Based on semi-quantitative RT-PCR, which was performed using nine 3B-specific primer pairs, four (CL24323Contig1, CL40247Contig1, CL35206Contig1, and CL23073Contig1) were not expressed, two (CL22200Contig1 and CL27257Contig1) were down-regulated and transcribed, and three, including CL29051Contig1, CL53819Contig1, and CL51762Contig1, were up-regulated and expressed in TA3575 as compared to CS (Fig. 3, Supplementary Fig. S3). On the other hand, genes not located on chromosome 3B, CL53958Contig1 and CL78654Contig1 were down-regulated and expressed, and CL23519Contig1 was up-regulated in TA3575, compared with CS. The results of both PCR amplifications using gDNA and semi-quantitative RT-PCR using RNA as templates were consistent with the RNA-seq data analyses and suggested that RNA-seq analyses in this study were valid and reproducible.

### Distribution of DEGs in TA7548

**Figure 4:**
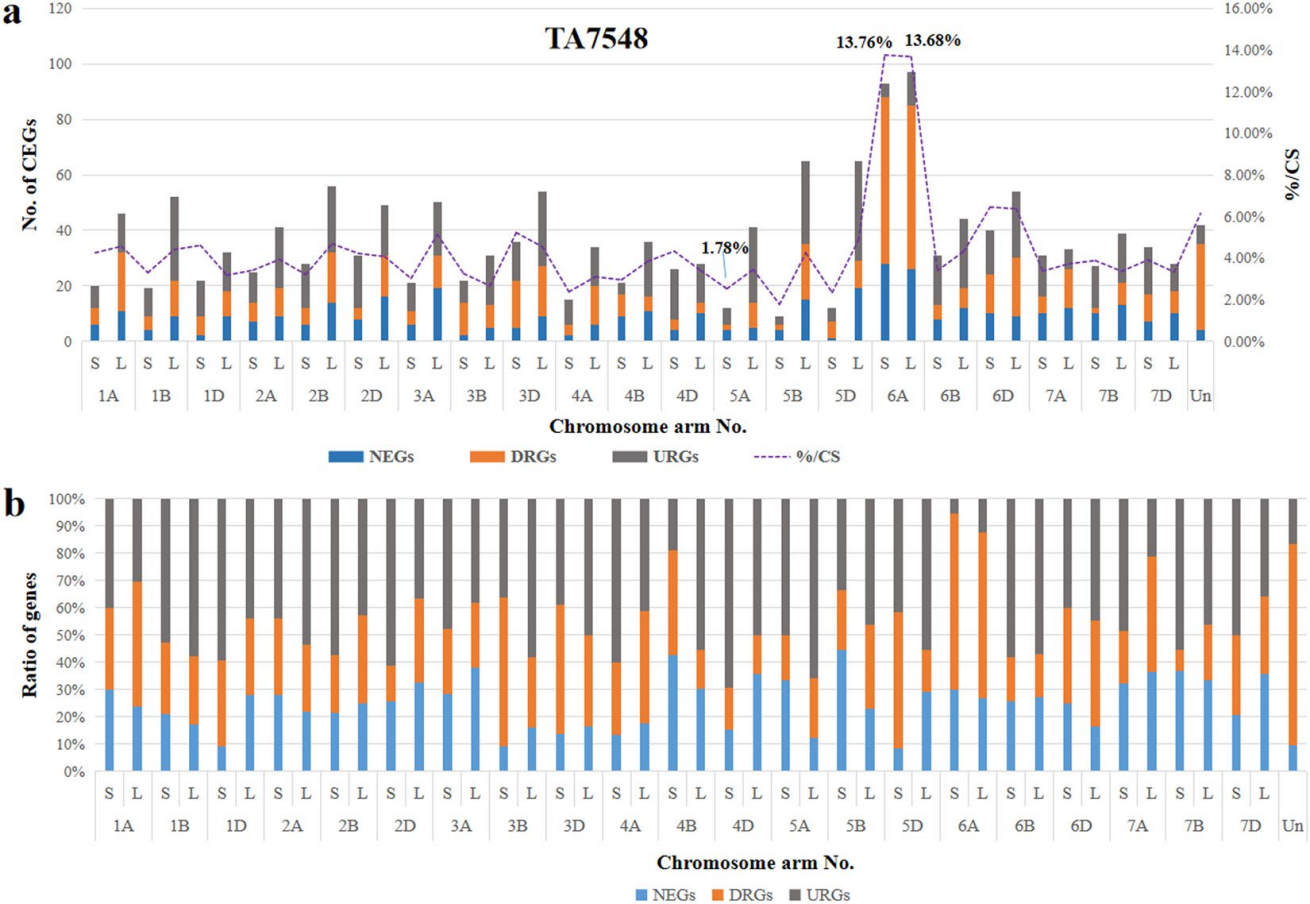
Chromosome distribution of DEGs in TA7548. (**a)** Distribution of DEGs on different chromosome arms. (**b)** Ratios of different DEGs on chromosome arms.

A total of 1,592 (4.3%) DEGs, including 396 NEGs (24.87%), 526 DRGs (33.04%) and 670 URGs (42.09%), were identified based on a pairwise comparison of expressed genes in CS-*Ae*. *longissima* DA 6S^l^#3 line TA7548 and CS on comparison of 37,058 mapped genes (Supplementary Tables S2, S3). These 1,592 DEGs were also asymmetrically distributed on different chromosomes in TA7548. The least affected genes were those located on the short arm of 5B; only nine out of 506 genes (1.78%) showed a change in transcription in TA7548 compared to that in CS. On the contrary, chromosome 6A of wheat contained the most DEGs (190/1,385, 7.50%), which included 54 (54/190, 28.42%) NEGs, 119 (119/190, 62.63%) DRGs and 17 (17/190, 8.95%) URGs (Fig. 4, Supplementary Table S2). On an average, about 90% of the DEGs were down-regulated or non-transcribed on both the short and the long arms of chromosome 6A, followed by chromosome 4BS, where more than 80% of DEGs were negatively regulated. In contrast, close to 70% of DEGs on the short arm of chromosome 4D were positively impacted by chromosome 6S^l^#3 in TA7548 (Fig. 4, Supplementary Table S2).

Unlike in TA3575, where the highest ratio of non-transcribed to transcribed genes, were those genes that were located to the missing chromosome 3B. The percentage of DRGs mapped to chromosome 6A in TA 7548 was significantly higher than that for any other chromosomes. The DEGs included 13.76% on the short arm and 13.65% on the long arm of chromosome 6A (Fig. 4). In addition, down-regulated genes (DRGs) were a major DEG type on both arms of chromosome 6A (Fig. 4), indicating that genes on both arms were negatively affected to the some extent by introgressed chromosome 6S^l^#3 in TA7548.

### Transcription-affected genes shared by both introgressed 3S^l^#2 and 6S^l^#3 in CS

Based on a pairwise comparison of DEGs between TA3575 and TA7548, 561 DEGs were simultaneously impacted by introgression of both chromosomes 3S^l^#2 and 6S^l^#3, which accounted for 41.25% and 38.79% of the total, excluding those on chromosome 3B in TA3575 (1,360) and 6A in TA7548 (1,446). Of the 561 shared DEGs, the overwhelming majority of genes (99.29%, 557/561) were consistently transcribed in both TA3575 and TA7548, with 84 (14.97%) not expressed, 60 (10.70%) down-regulated, and the majority of 413 up-regulated, accounting for 73.62% shared genes (Supplementary Table S3). Only four DEGs (0.71%, 4/561), including CL120979Contig1, CL14430Contig1, CL60355Contig1 and CL77981Contig1, were transcribed with different expression patterns between TA3575 and TA7548 (Supplementary Table S3). It is worth noting that the ratio of shared URGs to total shared DEGs (413/561, 73.62%) was much higher than that of URGs/DEGs in both TA3575 (722/1414, 51.06%) and TA7548 (665/1499, 44.36%).

**Figure 5:**
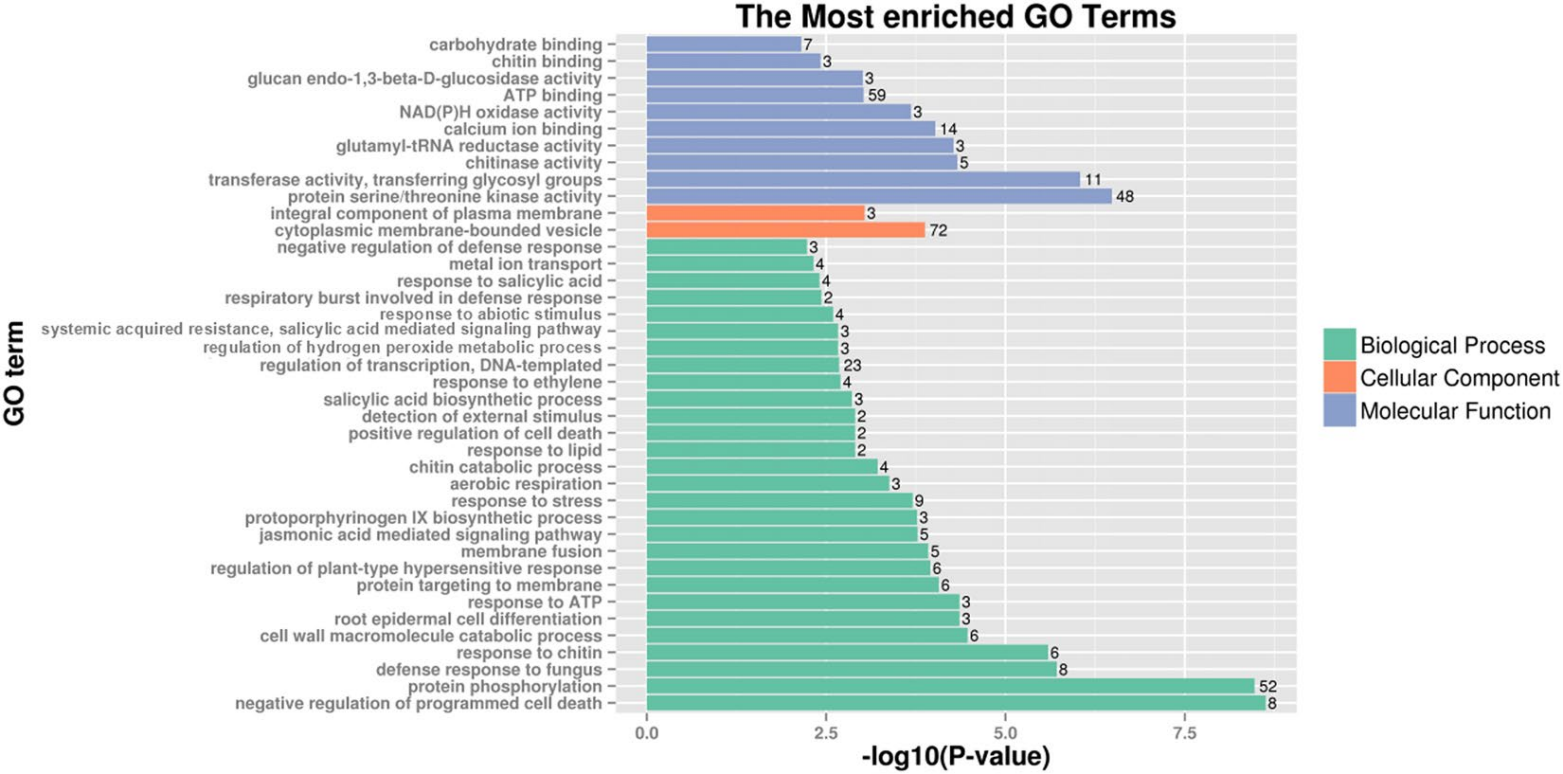
Go enrichment of DEGs shared by both 3S^l^#2 in TA3575 and 6S^l^#3 in TA7548. P-value = number of genes/the total genes annotated to a particular GO term in the whole genome. Only 40 pathways with the highest Go enrichment confidence P-value were represented. The number on the right of each lane in the figure represents the number of genes in the pathway.

The 561 DEGs shared by chromosomes 3S^l^#2 and 6S^l^#3 from *Ae*. *longissima* were annotated by blastx alignment against the GO database. The results indicated that most of the enriched, shared DEGs were categorized biologically as negative regulators of programmed cell death, followed by those involved in protein phosphorylation, defense response to fungus and response to chitin. Of those categorized as cellular components, cytoplasmic membrane-bounded vesicle and an integral component of the plasma membrane were the top terms. The molecular function category included protein serine/threonine kinase activity and transferase activity (Fig. 5, Supplementary Table S5).

### Impact of introgressed chromosomes 3S^l^#2 and 6S^l^#3 on putative disease resistance genes of CS

Both chromosomes 3S^l^#2 and 6S^l^#3 from *Ae*. *longissima* carry resistance gene(s) to powdery mildew of wheat^41^. The impact of 3S^l^#2 in TA3575 and 6S^l^#3 in TA7548 on the transcription of CS-derived putative plant disease resistance (R) genes was analyzed in this study. A sequence homology search of DEGs in the PRGdb discovered 34 (1.71%) R genes in seven classes from 1,991 DRGs in TA3575, with the maximum number of R genes in the RLP class (16, 47.06%), followed by those in classes NL (8, 23.53%), CNL and T (3, 8.82% each), and N, TNL and other (1, 2.94% each) (Table 2, Supplementary Table S6). Of these 34 putative R genes, 26 (76.47%) were up-regulated and eight (23.53%) down-regulated by chromosome 3S^l^#2. There were also two up-regulated R genes (RLP class) (CL77976Contig1 and CL29051Contig1) mapped on chromosome 3B (in CS) in TA3575 where chromosome 3B was missing, indicating that these two R genes are derived from 3S^l^#2 rather than CS wheat chromosome 3B (Table 2, Supplementary Table S6).

**Table 2:** List of 17 R genes affected by by the introgression of both 3S^l^#2 and 6S^l^#3 from *Ae. longissima*.

Gene id	R gene_id	Class	Description	Chromosome	Transcription
CL29244Contig1	PRGDB00189685	NL	*Hordeum vulgare* subsp. *vulgare* mRNA for predicted protein	chr1BL	Up
CL30613Contig1	PRGDB00167710	TNL	heat shock cognate protein 70-1	chr4BL	Up
CL34201Contig1	PRGDB00070731	T	*Malus x domestica* AP2 domain class transcription factor	chr2AL	Up
CL34201Contig2	PRGDB00070731	T	*Malus x domestica* AP2 domain class transcription factor	chr2AL	Up
CL50404Contig1	PRGDB00070731	T	*Malus x domestica* AP2 domain class transcription factor	chr3DL	Up
CL51766Contig1	PRGDB00189849	RLP	*Hordeum vulgare* subsp. *vulgare* mRNA for predicted protein	chr4AL	Up
CL5706Contig3	PRGDB00193023	NL	TSA: *Triticum aestivum* cultivar Bobwhite isotig12135	chr4BS	Up
CL58014Contig1	PRGDB00189776	Other	*Hordeum vulgare* subsp. *vulgare* mRNA for predicted protein	chr3DS	Up
CL61918Contig1	PRGDB00189228	NL	*Hordeum vulgare* subsp. *vulgare* mRNA for predicted protein	chr2BL	Up
CL858Contig2	PRGDB00077816	NL	*Triticum aestivum* cDNA, clone: WT007_G12	chr2BS	Up
comp11485_c0_seq. 1_2	PRGDB00189372	RLP	*Hordeum vulgare* subsp. *vulgare* mRNA for predicted protein	chr5BL	Up
comp22293_c0_seq. 1_3	PRGDB00204086	CNL	—	chr5AL	Up
CL34182Contig1	PRGDB00070031	RLP	*Hordeum vulgare* subsp. *vulgare* cDNA clone: FLbaf17p21	chr2BL	Down
CL80133Contig1	PRGDB00193641	RLP	TSA: *Triticum aestivum* cultivar Bobwhite isotig21593	chr6AL	Down
CL879Contig4	PRGDB00192952	RLP	TSA: *Triticum aestivum* cultivar Bobwhite isotig13214	chr1AL	Down
CL91059Contig1	PRGDB00193288	RLP	TSA: *Triticum aestivum* cultivar Bobwhite isotig09320	chr5DL	Down
comp18291_c0_seq. 1_4	PRGDB00192952	RLP	TSA: *Triticum aestivum* cultivar Bobwhite isotig13214	chr1AL	Down

CNL: contains a central nucleotide-binding (NB) subdomain, a leucine rich repeat (LRR) domain, and a predicted coiled-coil (CC) structures. NL: contains NBS and LRR domains, and lack of CC domain. RLP: contains leucine-rich receptor-like repeat, a transmembrane region of 25AA, and a short cytoplasmic region. TNL: contains a central NB subdomain, a LRR domain, and a interleukin-1 receptor (1L-1R) domain. T: contains TIR domain only, lack of LRR or NBS. Other: consists in a miscellaneus set of R proteins that do not fit into any of the known four classes, but that has resistance function. To date 13 genes were cloned to that class indicating that there are other way to produce resistance in plant kingdom.

In TA7548, 34 (34/1,592, 2.14%) DEGs were annotated as R genes, which were grouped in six classes, including CNL (6), NL (6), other (1), RLP (16), T (3) and TNL (2) R genes. Of these R genes, five in class CNL and 12 in class RLP were down-regulated; the 17 other R genes were up-regulated in TA7548 (Table 2). Three of the 34 R genes (CL33063Contig1, CL80133Contig1, and CL69182Contig1) were located on chromosome 6A, the CL33063Contig1 was up-regulated, whereas, the remaining two down-regulated in TA7548 that carries chromosome 6S^l^#3 (Supplementary Table S6).

A pairwise comparison of effected R genes between TA3575 and TA7548 further revealed that 17 R genes were impacted together by the introgression of both chromosomes 3S^l^#2 and 6S^l^#3. These 17 shared R genes were grouped into six classes, including one R gene for each class CNL, TNL and others, four in NL, seven in RLP and three in class T. Twelve (70.59%) R genes were transcribed with up-regulation, whereas the remaining five (29.41%), which all belong to class RLP, were down-regulated in both TA3575 and TA7548 (Supplementary Table S6). Transcription patterns of these 17 shared R genes were similar in both TA3575 and TA7548.

### Identification of a CS-*Ae. longissima* DS 6S^l^#3(6A) line from TA7548

As shown in TA3575, if a pair of wheat chromosomes is missing, non-transcribed genes account for the majority of DEGs located on the substituted chromosomes. In TA3575, where a pair of 3B chromosomes is replaced by a pair of 3S^l^#2 chromosomes, 461 out of 577 DEGs located to 3B were NEGs, accounting for as high as 79.90%. However, in TA7548 an unusually high ratio of DRGs (119/190, 62.63%) rather than NEGs (54/190, 28.42%) of chromosome 6A-specific DEGs led to the suspicion that chromosomes 6A might be missing in some TA7548 individuals (progenies) used for RNA-seq in this study.

**Figure 6:**
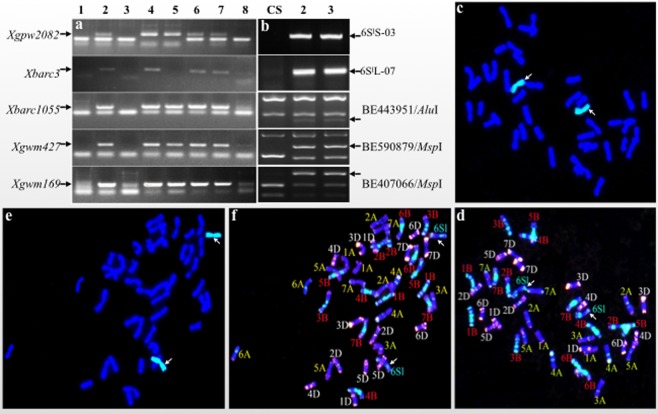
Identification of disomic 6S^l^#3(6A) substitution line in a TA7548 population. (**a**) PCR patterns of 6A-specific molecular markers of individuals in the TA7548 population. Arrows show the specific bands of chromosome 6A that are missing in plants 3 and 8. 1. N6A-T6B; 2–8. numbers of the tested plants. (**b)** PCR patterns of molecular markers of 6S^l^#3 from *Ae*. *longissima*. Arrows show the specific amplification bands of chromosome 6S^l^#3. (**c,d)** GISH and FISH patterns of a spontaneous disomic 6S^l^#3(6A) substitution line. Chromosomes 6S^l^#3 were painted green, and wheat chromosomes by blue in GISH photo. Arrows indicate chromosome 6S^l^#3 pairs. (**e,f**) GISH and FISH patterns of disomic 6S^l^#3(6A) addition line. Arrows show a pair of chromosome 6S^l^#3 painted green in GISH photo.

Molecular markers analysis of seven, randomly selected individual plants from TA7548 using five chromosome 6A-specific SSR and five chromosome 6S^l^-specific markers showed that all these chromosome 6A-specific SSR markers were present in five of the seven plants (5/7, 71.43%); two individuals (2/7, 28.57%) presented 6S^l^-specific markers but lacked 6A-specific markers (Fig. 6a,b, Supplementary Fig. S4). Subsequent genomic *in situ* hybridization (GISH) analysis of these individual plants indicated that the two individuals missing 6A-specific markers had 42 chromosomes, 40 wheat chromosomes plus a 6S^l^ chromosome pair. Further fluorescent *in situ* hybridization (FISH) analysis verified that a pair of 6A chromosomes was replaced by a 6S^l^#3 chromosome pair in these two plants, which resulted in a disomic 6S^l^#3(6A) substitutions (Fig. 6c,d). The remaining five plants were confirmed to be disomic 6S^l^#3 addition lines, which had 42 wheat chromosomes plus a pair of 6S^l^ chromosomes (Fig. 6e,f).

Therefore, the TA7548 population used in this study was composed of about 71.43% DA 6S^l^#3 and 28.57% DS 6S^l^#3(6A) plants. This novel CS-*Ae*. *longissima* DS 6S^l^#3(6A) line, spontaneously formed during the long process of reproduction of the original DA 6S^l^#3 plants, was named TA7548-6A. The substitution of chromosome 6A by chromosome 6S^l^#3 in some individuals of TA7548 led to the unusually high ratio of DRGs of chromosome 6A in TA7548 used for RNA-seq in this study.

## Discussion

Linkage drag, caused by alien chromosome segments introduced into wheat genetic backgrounds, has led to unfavorable agronomic and end-use quality traits and, thus, limited the utilization of alien genes in wheat improvement programs. The linkage drag caused by deleterious genes associated with targeted genes had been widely demonstrated^21,50–52^. However, linkage drag that may be attributed to the inability of alien genes to replace missing wheat genes, or the interactions between the introduced alien genes and recipient wheat genes, has, until now, been relatively under-researched^38,53^. In this study, we verified the genome-wide impact on wheat gene expression by the presence of introduced alien chromosomes. Transcription of 1,413 (4.01%) of 35,219 genes mapped to non-3B chromosomes in CS were significantly changed by the substitution of a pair of chromosome 3B by chromosome 3S^l^#2 derived from *Ae*. *longissima* in TA3575. Whereas 1,402 (3.93%) of 35,673 non-6A genes in CS were affected by introgression of chromosome 6S^l^#3 in TA7548. The ratios of affected genes in this study were higher than the 3% (960/35,301) reported by Rey *et al*. (2018), investigating the effect of introgression of barley telosomic 7HL in CS^38^. Further analyses of DEGs on different wheat chromosomes demonstrated that the impact of introgressed chromosomes 3S^l^#2 and 6S^l^#3 was obviously different for wheat chromosomes. The minimum ratio of DEGs was associated with chromosome 5AS in TA3575 (1.47%) and chromosome 5BS in TA7548 (1.78%); whereas the highest ratios were on chromosomes 3DS in TA3575 (7.55%) and 6DL in TA7548 (6.35%). Those negatively impacted genes, including non-transcribed and down-regulated, accounted for 48.90% (691/1,413) in TA3575, and 53.42% (749/1,402) in TA7548. Whether these impacts may lead to linkage drag of agronomic traits, or the effects can be decreased in different recipient wheat backgrounds, are currently under investigation. The present study will benefit the understanding of gene interactions between recipient wheat and the alien donor species.

Of those DEGs caused by introgressed chromosomes 3S^l^#2 and 6S^l^#3 from *Ae*. *longissima*, 17 genes in six R gene classes were annotated as putative R genes from 561 DEGs shared by introgressed 3S^l^#2 in TA3575 and 6S^l^#3 in TA7548. Transcription analysis of these 17 R genes, based on transcriptome analyses, showed that 12 shared R genes (70.59%) were significantly up-regulated and transcribed in both TA3575 and TA7548, whereas only five genes (29.41%) in class RLP were down-regulated. Because both chromosomes 3S^l^#2 and 6S^l^#3 confer resistance to powdery mildew of wheat^41^, whether the transcription changes of these shared R genes were involved in the pathogen-defense processes deserved to be further investigated. Molecular marker could be developed from these R gene for mapping of the two powdery mildew resistance genes introgressed from *Ae*. *longissima*.

RNA-sequencing techniques have provided a very useful means for examining chromosome structural changes^38,54,55^. In this study, the discovery of an abnormally high percentage of DRGs on both the short and the long arm of wheat chromosome 6A, based on RNA-seq analyses, implied that chromosome variation of chromosome 6A might exist in the CS-*Ae*. *longissima* DA 6S^l^#3 line TA7548. We further verified that 28.57% of the plants in the TA7548 population were, in fact, spontaneously formed DS 6S^l^#3(6A) by integration analyses of RNA-seq data, molecular markers and *in situ* hybridization. This novel DS 6S^l^#3(6A) line (TA7548-6A) will be useful for further transfer of 6S^l^#3 resistance genes to powdery mildew into wheat by inducing wheat-*Ae*. *longissima* 6S^l^-6A translocations. In a similar way, we selected a spontaneous Robertsonian translocation T4S^l^#2·4BL carrying the powdery mildew resistance gene *Pm66*^56^. In summary, integrated use of genome sequencing, molecular markers, and classic cytogenetic techniques can speed up the introgression of alien targeted genes, thus promoting the utilization of wild relatives of wheat in breeding programs.

## Conclusion

In the current study, transcriptome analyses were performed using two CS-*Ae. longissima* introgression lines carrying powdery mildew resistance gene(s) and their genetic background line wheat CS. The results showed that introgression of chromosomes 3S^l^#2 and 6S^l^#3 derived from *Ae*. *longissima* had genome-wide impact on gene transcription in wheat. A total of 5.37% and 4.30% genes (or 4.01% of non-3B-specific genes and 3.93% of non-6A-specific genes) were differentially transcribed due to the introduction of chromosomes 3S^l^#2 in TA3575 and 6S^l^#3 in TA7548, respectively. Furthermore, 17 putative R genes of wheat were significantly impacted together in both TA3575 and TA7548, which carry chromosomes 3S^l^#2 and 6S^l^#3. The majority of these shared R genes (70.59%) were significantly up-regulated by introgression of chromosomes 3S^l^#2 and 6S^l^#3. Whether these shared putative R genes were involved in the defensive processes initiated by powdery mildew resistance genes located on chromosomes 3S^l^#2 and 6S^l^#3 needs to be further investigated.

## Materials and methods

### Plant materials

Wheat landrace Chinese Spring (CS); nullisomic 3B-tetrasomic 3D (N3B-T3D, TA3272), where a pair of chromosome 3B is replaced by two pairs of 3D; N6A-T6B (TA3152) where 6A is substituted by two pairs of 6B; a CS-*Ae*. *longissima* disomic 3S^l^#2(3B) substitution line TA3575, where a pair of 3S^l^#2 chromosomes replaces the 3B chromosome pair in CS; and a CS-*Ae*. *longissima* disomic 6S^l^#3 addition line TA7548, where a pair of 6S^l^#3 chromosomes are added to the chromosome complement of CS; were used in this study. Both chromosomes 3S^l^#2 and 6S^l^#3 confer resistance to powdery mildew^41^. The number following the chromosome designation is used to distinguish between the same *Ae. longissima* chromosome derived from different *Ae. longissima* accessions^57^. All plant materials were kindly provided by the Wheat Genetic Resources Center (WGRC) at Kansas State University, USA, propagated and stored at the experimental station of Henan Agricultural University.

### Plant growth and tissue collection

Seeds of the two wheat-*Ae*. *longissima* introgression lines (TA3575 and TA7548) and CS were sterilized with 5% sodium hypochlorite solution for 10 min at room temperature and then planted in 8-cm diameter pots filled with an autoclaved vermiculite potting medium. The seedlings were grown in an illuminated incubator at 18–20 °C, 18 h light and 6 h dark, and 75% relative humidity. Pooled, equal-length segments from the first leaf of 10 individuals for each line were then immediately frozen in liquid nitrogen for subsequent RNA isolation. Two independent, biological replicates were collected for subsequent cDNA library construction and RNA sequencing.

### Construction of cDNA libraries for Illumina sequencing

Total RNA was extracted from leaf samples of three plants using an mirVana miRNA Isolation Kit (Cat. No. AM1561, Ambion, Thermo Fisher Scientific Inc., Waltham, MA, USA) following the manufacturer’s protocol. RNA integrity was evaluated using an Agilent 2100 Bioanalyzer (Agilent Technologies, Santa Clara, CA, USA). The samples with an RNA Integrity Number (RIN) ≥ 7 were subjected to subsequent analysis. The libraries were constructed using TruSeq Stranded mRNA LTSample Prep Kit (Illumina, San Diego, CA, USA) according to the manufacturer’s instructions. These libraries were sequenced on the Illumina sequencing platform (HiSeqTM 2500 or Illumina HiSeq X Ten) by OE Biotech (Shanghai), China, and 125 bp/150 bp paired-end reads (raw reads) were generated.

### RNA-seq data analysis

Paired-end reads were pretreated using the NGS QC Toolkit software to remove sequences containing adapters or poly-N above 5%, and low-quality reads to produce valid data (clean data) for downstream sequence assembly^58^. Valid ratio, Q30 and GC content of each sample were calculated after pretreatment.

*De novo* assembly of clean data was applied using Trinity (version: trinityrnaseq_r20131110)^59^, followed by the removal of abundant sequences using TGICL^60^. Annotation and function of these genes were assigned with a cut off e-value <1e^−5^ based on blastx alignment against protein sequences in public databases NR (NCBI non-reduction protein sequences, ftp://ftp.ncbi.nih.gov/blast/db), GO (Gene Ontology, http://www.geneontology.org) and PRGdb (Plant Resistance Gene Database)^61^.

For DEGs analysis, the read counts of genes were first normalized as FPKM. Then, the expression significance of genes of CS vs. TA3575 and CS vs. TA7548 were calculated using DESeq (http://bioconductor.org/packages/release/bioc/html/DESeq.html) with a threshold FDR ≤ 0.05 & |log_2_(fold change)| ≥ 1. Genes with an FPKM ≥ 0.5 were considered expressed, whereas genes with FPKM = 0 were not transcribed. Genes of CS were pair-wise compared with those of TA3575 and TA7548 to identify genes present in CS and absent in TA3575 and TA7548. All the genes were assigned to chromosome arms based on blastn alignment against wheat reference genomic sequences (CS RefSeq v1.0)^62^ with a cutoff of expect < =1e-10&qcov > =75% at UGRI BLAST (https://urgi.versailles.inra.fr/blast/blast.php).

### Molecular marker analysis

Genomic DNA (gDNA) was extracted from young leaf segments with a DNeasy Plant Mini Kit (Qiagen, Cat No. 69104) following the manufacturer’s directions. Total RNA was isolated using an RNA Prep Pure Plant Kit (Tiangen Biotech Co. Ltd., Code No. DP432), and cDNA synthesized using a PrimeScript™ Double Strand cDNA Synthesis Kit (Takara, Code No. 6111A).

In total, 44 molecular markers were used in the study (Supplementary Table S1). Of these, 10 3B-specific SSR and 12 3S^l^#2-specific EST markers were used for the karyotype analysis of TA3575^63,64^. Another nine PCR primer pairs of 3B-specific and three non-3B chromosomes were designed based on transcription sequences of CS and used for validation of gene expression changes in TA3575. The remaining five 6A-specific SSR and five 6S^l^#3-specific markers were used for molecular marker analyses of TA7548^63,65–67^. PCR reaction mixture preparation and amplification by “F50SSR” (for SSR markers) or “Touch-down 63” (for the remaining markers) followed Liu *et al*.^14^. The PCR products were digested with restriction enzymes followed by Liu *et al*.^14^. The PCR products or restricted fragments were resolved in 2.5% agarose gels and visualized by ethidium bromide staining under UV light.

### Chromosome preparation and *in situ* hybridization

Root tip collection, nitrous oxide treatment, fixation, and slide preparations were according to Liu *et al*.^14^. GISH using fluorescein-12-dUTP labeled genomic DNA of *Ae*. *longissima* as the probe, was followed the procedure of Liu *et al*.^14^. The ratio of genomic *Ae*. *longissima* DNA and CS blocking DNA was 1:120. FISH using FAM (6-carboxyfuorescein) and TAMRA (6-carboxytetramethylrhodamine) modified oligonucleotide probes followed Huang *et al*.^68^. After hybridization and slide washing, 25–30 µl of Vectashield mounting medium containing 1 µg/ml DAPI (Vector Laboratories Inc, Burlingame, CA, USA) was added to each slide and then covered with a 24 × 30 cm glass coverslip. Observation of fluorescent images was on a Zeiss Axio Scope A1 fluorescence microscope (Germany) and captured with an AxioCam MRc5 CCD camera. Images were further processed with Adobe Photoshop CS3 (Version 10.0.1) (Adobe Systems Inc., San Jose, CA, USA).

## Supplementary information

Supplementary Information.

Table S1.

Table S2.

Table S3.

Table S4.

Table S5.

Table S6.

Supplementary Figures.
