# Supplementary material for: Genome-wide impacts of alien chromatin introgression on wheat gene transcriptions
Source: Sci Rep. 2020 Mar 16;10:4801. doi: 10.1038/s41598-020-61888-1 (PMC7076028; doi:10.1038/s41598-020-61888-1)
Supplement: Supplementary file 1 — Supplementary Information. [file 41598_2020_61888_MOESM1_ESM.docx]

**Genome-wide impacts of alien chromatin** **introgression on wheat gene transcriptions**

Zhenjie Dong^1^ · Chao Ma^1^ · Xiubin Tian^1^ · Changtao Zhu^2^ · Gang Wang^2^ · Yuanfeng Lv^2^ · Bernd Friebe^3^ · Huanhuan Li^1*^ · Wenxuan Liu^1*^

^1^National Key Laboratory of Wheat and Maize Crop Science, College of Life Sciences, Henan Agricultural University, Zhengzhou, 450002, China

^2^Yu’An Institute of Wheat, Wen County, 454850, China

^3^Wheat Genetic Resources Center, Department of Plant Pathology, Throckmorton Plant Sciences Center, Kansas State University, Manhattan, KS, 66506, USA

^*^Corresponding authors:

Huanhuan Li E**-**mail: [huanhuanli.happy@163.com](mailto:huanhuanli.happy@163.com)

Wenxuan Liu E**-**mail: wxliu2003@hotmail.com
