# Supplementary figures and images for: Genome-wide impacts of alien chromatin introgression on wheat gene transcriptions
Source: Sci Rep. 2020 Mar 16;10:4801. doi: 10.1038/s41598-020-61888-1 (PMC7076028; doi:10.1038/s41598-020-61888-1)

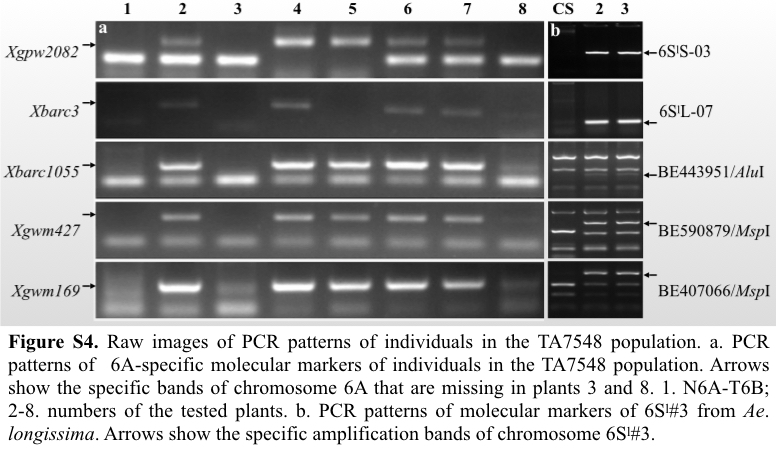

Supplement: Supplementary file 8 — Supplementary Figures. [file 41598_2020_61888_MOESM8_ESM.zip › Supplementary Figures/Supplementary Fig. S4.jpg]

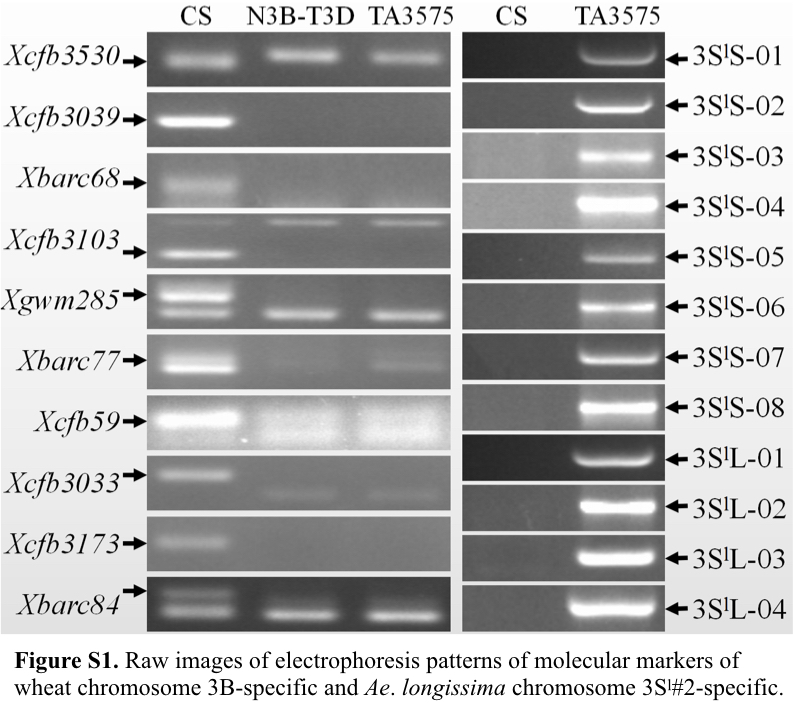

Supplement: Supplementary file 8 — Supplementary Figures. [file 41598_2020_61888_MOESM8_ESM.zip › Supplementary Figures/Supplementary Fig. S1.jpg]

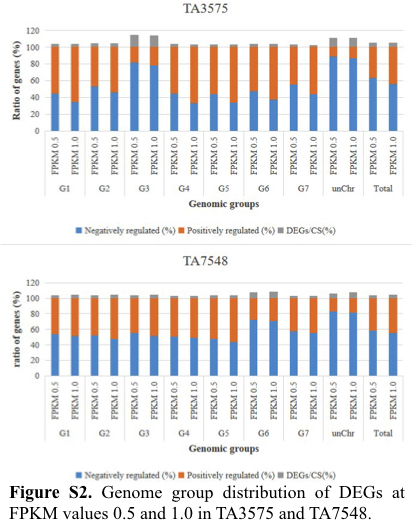

Supplement: Supplementary file 8 — Supplementary Figures. [file 41598_2020_61888_MOESM8_ESM.zip › Supplementary Figures/Supplementary Fig. S2.jpg]

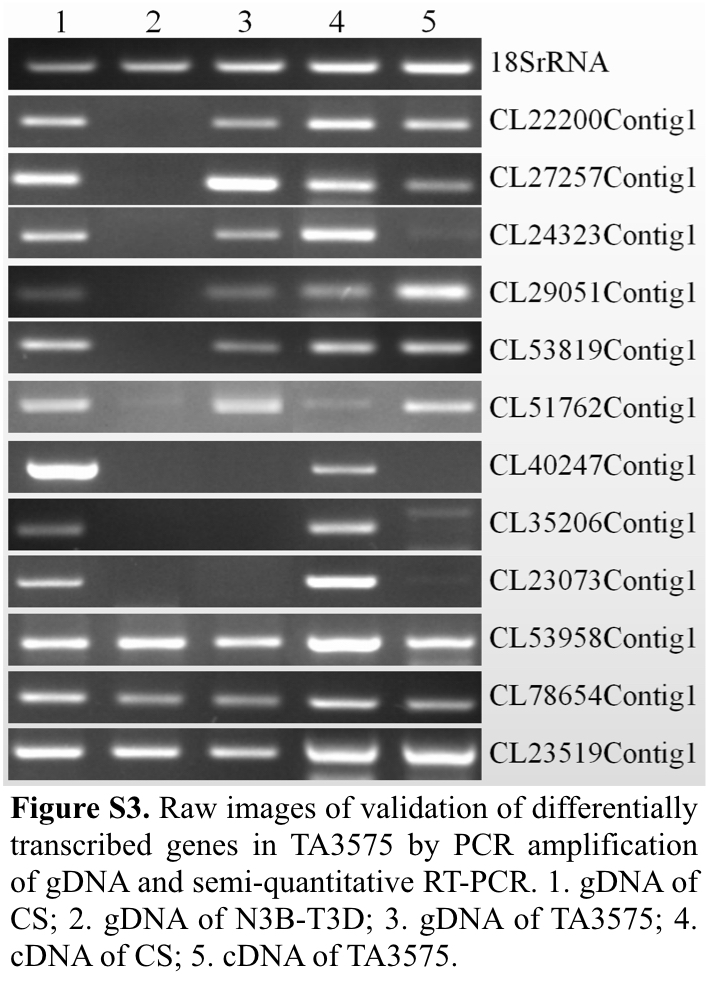

Supplement: Supplementary file 8 — Supplementary Figures. [file 41598_2020_61888_MOESM8_ESM.zip › Supplementary Figures/Supplementary Fig. S3.jpg]
